# Cxs and Panx- hemichannels in peripheral and central chemosensing in mammals

**DOI:** 10.3389/fncel.2014.00123

**Published:** 2014-05-09

**Authors:** Edison Pablo Reyes, Verónica Cerpa, Liliana Corvalán, Mauricio Antonio Retamal

**Affiliations:** ^1^Centro de Fisiología Celular e Integrativa, Facultad de Medicina, Clínica Alemana Universidad del DesarrolloSantiago, Chile; ^2^Dirección de Investigación, Universidad Autónoma de ChileSantiago, Chile

**Keywords:** gap junctions, carotid body, glomus cells, connexins, astrocytes, hypoxia, hypercapnia

## Abstract

Connexins (Cxs) and Pannexins (Panx) form hemichannels at the plasma membrane of animals. Despite their low open probability under physiological conditions, these hemichannels release signaling molecules (i.e., ATP, Glutamate, PGE_2_) to the extracellular space, thus subserving several important physiological processes. Oxygen and CO_2_ sensing are fundamental to the normal functioning of vertebrate organisms. Fluctuations in blood PO_2_, PCO_2_ and pH are sensed at the carotid bifurcations of adult mammals by glomus cells of the carotid bodies. Likewise, changes in pH and/or PCO_2_ of cerebrospinal fluid are sensed by central chemoreceptors, a group of specialized neurones distributed in the ventrolateral medulla (VLM), raphe nuclei, and some other brainstem areas. After many years of research, the molecular mechanisms involved in chemosensing process are not completely understood. This manuscript will review data regarding relationships between chemosensitive cells and the expression of channels formed by Cxs and Panx, with special emphasis on hemichannels.

## Introduction

**Figure 1:**
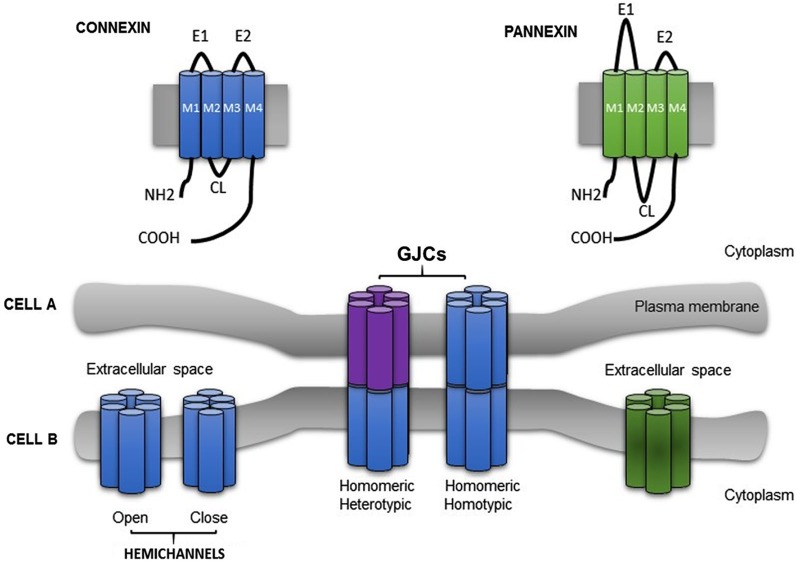
**Schematic illustration showing the topological structure of connexin and pannexin**. Six connexins (Cxs) (a protein with four membrane domains (M1–M4), two extracellular loops (E1, E2), one cytoplasmic loop (CL) and the N and C termini exposed to the cytoplasm) subtype oligomerize into homomeric or heteromeric hemichannel. Under physiological circumstances, hemichannel remain closed. Gap junction channels (GJCs) (homotypic or heterotypic) connect the cytoplasm of two adyacent cells (Cell A and Cell B), allowing the passage of a variety of small molecules. Pannexin showed a similar topological structure.

In vertebrates, the family of membrane proteins that forms gap junction channels (GJCs) is called connexin. To date, 21 connexin isoforms in the human genome and 20 in the mouse genome (Willecke et al., 2002) have been described in almost all cell types, except for in mature sperm cells, differentiated skeletal muscle (in physiological conditions) and erythrocytes. The topological organization of the connexin-protein family is highly preserved and consist of four transmembrane regions linked by one intercellular cytoplasmic loop (CL), two extracellular loops (E1 and E2) (Hertzbergs et al., 1988; Milks et al., 1988; Falk et al., 1994; reviewed by Bruzzone et al., 1996; Kumar and Gilula, 1996) and both protein termini are located at the cytoplasmic side. The C-terminus length is variable and it is subjected to post-translational modifications related to intracellular signal cascades (Sáez et al., 1998; Figure 1). Connexins are abbreviated “Cx” followed by the molecular mass in kDa, e.g., Cx43 (Beyer et al., 1987). However, an alternative nomenclature categorized Cxs into five different groups: α, β, γ, Δ and ϵ based on homology (specifically “the extent of sequence identity”) and length of their CL (Bennett et al., 1991; Nielsen et al., 2012).

The expression of Cxs has a distinctive spatial, temporal and overlapping pattern (reviewed by Oyamada et al., 2005; Rackauskas et al., 2010) and the physiological relevance of different Cxs has been studied using several approaches (Cx knock-out animals, specific mutations in Cx genes and down regulating or changing the expression pattern of GJC). These studies showed that, in different organs, the disruption of GJCs can lead to pathological conditions such as, cataract formation, epidermal disease, hearing loss, apoptosis or cancer (Baruch et al., 2001; Saito et al., 2001; Common et al., 2005; Aishah et al., 2008; Kameritsch et al., 2013).

Cxs oligomerize forming aqueous hexameric hemichannels called connexons. Oligomerization occurs in intracellular compartments depending on the connexin type, e.g., Cx43 assemble in the trans-Golgi network (Musil and Goodenough, 1993; George, 1999) and Cx32 in the endoplasmic reticulum (Das Sarma et al., 2002). Connexons can be built of one or different Cxs isoforms assembling homomeric or heteromeric hemichannels, respectively. GJCs result from the association of two hemichannels, each provided by one of the two participating cells (Perkins et al., 1997; Unger et al., 1999). A connexon may dock with either, an identical or a different hemichannel forming homotypic or heterotypic channels, respectively (Kumar and Gilula, 1996). Henceforth, four arrangements of channels are possible (Figure 1). As the majority of cells expresses more than one Cx isoform, channels formed by heteromeric connexons should be the obvious study matter. However, since Cxs have different intracellular pathways to oligomerize, and at least two different pathways to be transported to the plasma membrane (George, 1999; Martin et al., 2001), the research focused on homomeric heterotypic channels (Werner et al., 1989; Elfgang et al., 1995; Falk et al., 1997; Gemel et al., 2004).

Gap junction (GJs) are clusters of intercellular channels (GJCs) present in almost all cell types. Initially, GJCs were described as nonspecific passive pores permeable to all soluble second messengers (e.g., amino acids, nucleotides, Ca^2+^, glucose and metabolites smaller than 1.2 kDa) (reviewed by Bruzzone et al., 1996). These channels provide cytoplasmic connections between two adjacent cells allowing the exchange of signaling molecules (ions, second messengers and small metabolites) (reviewed by Bennett et al., 1991; Bruzzone et al., 1996; Goodenough et al., 1996). This direct cell to cell electric and metabolic communication is essential in many physiological processes (e.g., embryonic development, propagation of action potential, cell growth and differentiation), synchronizing the function of organs including heart, liver, testis, skin and brain.

Hemichannels were considered a non-functional part of an intercellular communication pore. The rationale was: since GJCs are nonspecific if hemichannels were open at the plasma membrane, important components of the cytoplasm may “leak” to the extracellular medium and the cell would have to spent enormous amounts of energy to maintain its homeostasis. Today it is well known that “functional hemichannels” expressed in non-junctional plasma membrane of several cell types providing direct communication between intra- and extra-cellular environments (reviewed in Sáez et al., 2003, 2005). Under normal circumstances, hemichannels are closed and maintains cells isolated from external conditions. They become open after membrane depolarization, extracellular alkalization, metabolic inhibition, mechanical stimulation or in low extracellular calcium (DeVries and Schwartz, 1992; Ebihara et al., 1995; John, 1999; Contreras et al., 2002; Retamal et al., 2006; Schalper et al., 2010). Interestingly, removal of extracellular calcium in isosmotic condition also induce reversible changes in the cellular volume of different cells that normally express Cxs (e.g., fibroblast, endothelial and epithelial cells) (Quist et al., 2000). Even more, studies performed in *Xenopus* oocytes showed that hemichannels act as cationic channels, having distinctive voltage-dependent properties (reviewed by Bukauskas and Verselis, 2004).

Once functional hemichannels opened, they release NAD+, ATP, glutamate and prostaglandin E_2_ to the extracellular space (Bruzzone et al., 2001; Stout et al., 2002; Ye et al., 2003; Cherian et al., 2005). These molecules play a critical role in central nervous system (CNS) physiology, hepatic homeostasis, and several paracrine/autocrine signaling (Corriden and Insel, 2010; Vinken, 2011; Orellana et al., 2013; Wang et al., 2013a). Pathological situations, such as oxidative stress or metabolism inhibition, may also open hemichannels allowing the movement of above molecules, which contribute to cell damage activating apoptotic mechanisms or altering cell physiology (Lin et al., 2003; Retamal et al., 2006; Ramachandran et al., 2007; Schalper et al., 2008).

Recently, it has been identified a novel family of integral membrane proteins, which share some structural and functional characteristics with Cx: pannexins (Panxs; Panchin et al., 2000; Yen and Saier, 2007; Sosinsky et al., 2011). Panxs are encoded by three genes: pannexin 1 (Panx1); pannexin 2 (Panx2) and pannexin 3 (Panx3), showing a 50–60% of sequence similarity (Sosinsky et al., 2011). Topologically, Cx and Panx have the same structure (four transmembrane segments, cytoplasmatic termini and two extracellular loops; Figure 1). Panx1 is ubiquitously expressed (e.g., brain, kidney, liver, retina, testis, skeletal and heart muscle, etc.), Panx2 is predominantly expressed in CNS and Panx3 is expressed in embryonic tissue, osteoblast and synovial fibroblast (Panchin et al., 2000; Bruzzone et al., 2003; Baranova et al., 2004). Functional studies performed in *Xenopus* oocytes demonstrated that Panx could be expressed in non-junctional membranes, forming hemichannels referred to as pannexons. When these opened, they allow the uptake and/or release of metabolites such as Ca^2+^, anions and ATP (Vanden et al., 2006; Ambrosi et al., 2010; Ma et al., 2012; Romanov et al., 2012).

It has been described that pannexons from adjacent cells, may dock forming intercellular channels, but this idea is still controversial (Bruzzone et al., 2003). Just like Cxs, Panxs could form homotypic (Panx1) and heterotypic (Panx1/Panx2) functional channels, but the latter are unstable and completely disaggregate after 24 h (Ambrosi et al., 2010). Panx3 have not been functionally expressed in these experimental approaches (Bruzzone et al., 2003).

The biological relevance of Panxs is well accepted. There are some studies revealing their participation in specific processes, e.g., skeletal muscle release ATP through Panx1 after repetitive stimulation (Riquelme et al., 2013; Valladares et al., 2013). Likewise, neurons—and possibly also astrocytes—release arachidonic acid derivatives through Panx1. Those derivatives may be involved in a novel way of calcium wave propagation (MacVicar and Thompson, 2010). Also Panx1 may be involved in the regulation of the vascular tone regulating the release of ATP throughout the arterial network (Billaud et al., 2011; Lohman et al., 2012). Interestingly, Panx1 appears to be involved in a novel tri or quadripartite synapse at the carotid body (CB) chemoreceptors, amplifying the ATP signaling (Zhang et al., 2012; Piskuric and Nurse, 2013). The biological function of Panx3 is not clear and remains to be studied, but it has been related with osteoblast differentiation by functioning as calcium channels at the endoplasmic reticulum (Ishikawa et al., 2011) and to the differentiation of keratinocytes of the epidermis (Celetti et al., 2010).

Considering all physiological processes in which Cx and Panx are involved and the fact that both proteins are expressed in chemosensory systems, this review will outline the key features related to their biological relevance in the homeostasis of PO_2_, PCO_2_ and pH.

Finally, the evidence suggests that GJCs in non-excitable tissue may contribute to the spread of calcium waves (Gomes et al., 2006).

## Cx and Panx channels in arterial chemoreception

**Figure 2:**
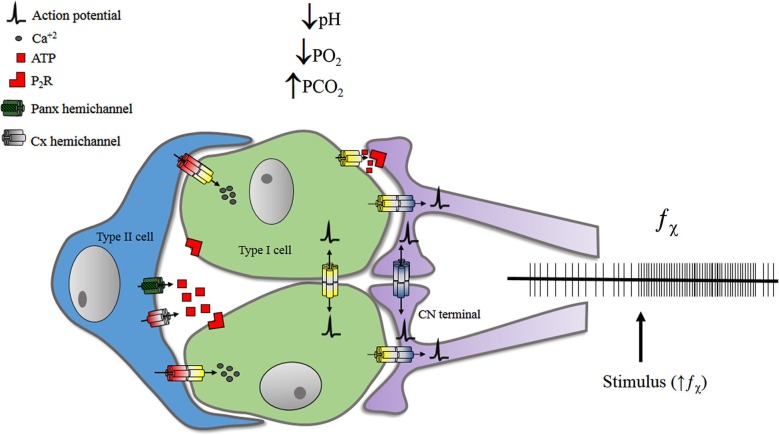
**Representation of some pathways proposed for f_bchi** increase upon stimulation. GJCs may participate on electrical chemosensory transmission from type I cells to CN terminals. Also GJCs may propagate electrical signals between glomus cells and/or between CN terminals. The release of ATP—from glomus and/or sustentacular cells—may also influence the chemosensory discharge of the carotid nerve. Details are discussed in the text. Chemical synapses are not represented.

The main peripheral chemoreceptor is the CB. It is located at the carotid bifurcation and innervated by the carotid nerve (CN), a branch of the glossopharyngeal nerve (IXth pair). The CB is a compound receptor, where clusters of chemosensory units—glomus (Type I) cells—are surrounded by sustentacular (Type II) cells, and are found in close contact with *en calyce* endings of CN (Figure 2).

Information of arterial PO_2_ and PCO_2_ is conveyed from glomus cells to nerve endings. Chemical synapses in the CB have been extensively studied and many transmitters have been described so far (for a review see Zapata, 1997a, b). However, the electrical transmission only appears as a possibility if Type I cells change its membrane potential. Glomus cells not only depolarize, but even more, they are capable of generating action potentials, either spontaneously (Duchen et al., 1988) or evoked by depolarizing currents (López-López et al., 1989). Therefore, the information sensed by Type I cells can be conveyed to CN endings by chemical and/or electrical synapses.

Early evidence of functional coupling at cat CB cells was provided by electrophysiological studies in impaled cells stained with procion navy blue for ulterior recognition, and the dye from these cells spread to others (Baron and Eyzaguirre, 1977). Above study availed the previously described ultrastructural evidence of GJCs between rat glomus cells (McDonald, 1976), as a “plausible explanation” for dye spreading. The species puzzle was solved when lucifer yellow injected into one cat carotid glomus cell spread to other cells (Chou et al., 1998). Finally, Cx43 was identified in rat carotid bodies using Western blots and immunocytochemical methods, clearing up doubts about this issue (Abudara et al., 1999).

Despite the functional evidence, the detection and identification of GJs in the CB was elusive. Controversial evidence was provided using different techniques, even though they were reported by the same group of researchers. Using freeze fracture analysis, Kondo and Yamamoto did not find the characteristic GJC clusters (Kondo, 1981; Kondo and Yamamoto, 1993). However, using freeze substitution after aldehyde-prefixation, they found GJ-like structures between Type I cells and Type I cells and nerve terminals (Kondo and Iwasa, 1996), accounting for the electro-coupling and also enlighten some unexplained data (McQueen and Evrard, 1990). McQueen used selective antagonists to study the role of transmitters in the CB chemotransmission. Although the pharmacological effects were blocked, the response evoked by physiological stimuli still remained. This controversy still persisted because the technique causes artifactual GJ-like structures (Kondo, 2002).

Despite above results, dye spreading from one cell to others occurs because GJCs are able to convey molecules; therefore, current spreading is also possible. This was tested by Eyzaguirre’s group (Monti-Bloch et al., 1993), impaling two adjacent Type I cells using independent amplifiers. To obtain the coupling coefficient (Bennett, 1966), they measured the membrane potential of both cells, while current was injected to one of them. They also calculated the coupling resistance (Spray et al., 1981), recording intracellular currents, while clamping the voltage at different potentials or during the application of chemical stimuli. Regarding glomus cells, current spreading from one cell to another (glomic coupling) has two characteristics: (1) coupling is bidirectional: the current spreads from one to another cell, no matter which cell is stimulated; (2) coupling is resistive: the response in the second cell is maintained during the stimulation of the first one (Jiang and Eyzaguirre, 2006; Eyzaguirre, 2007).

In eucapnia (normocapnic normoxia), the degree of coupling between glomus cells is variable and it seems to be reduced by CB stimulants such as acid, hypercapnia or hypoxia, in agreement with studies showing that these stimuli close GJC (Peracchia et al., 2003; Peracchia, 2004). Nevertheless, the uncoupling effect of chemosensory stimuli was not uniform, since the majority of coupled glomus cells reduced their coupling, but some are found more coupled (Monti-Bloch et al., 1993; Eyzaguirre and Abudara, 1995, 1999). The explanation for this irregular result considers that glomus cells uncouple for transmitter secretion—just like secretory cells at exocrine glands (for references see Bennett and Spray, 1985; Bennett et al., 1991), hence the uncoupling during stimulation. Also, the enhanced coupling of some cells during stimulation may be compatible with transmitters recharging or production by those cells (Eyzaguirre and Abudara, 1999). We consider that the explanation may be extrapolated for the coupling disparity in basal conditions. Taking into account that chemosensory discharge exists in eupneic condition and in the absence of CO_2_ (Eyzaguirre and Lewin, 1961; for a thorough discussion see Zapata, 1997b), it is very likely that some glomus cells were secreting transmitters in those conditions. Therefore, some of glomus cells will be uncoupled and some coupled, secreting and recharging transmitters, respectively.

Other remarkable observation is related to glomus cells depolarization. There seems to be a correlation between depolarization and uncoupling. On the one hand, glomus cells are known to be depolarized during transmitter release (Monti-Bloch et al., 1993). Although the transductional mechanism of chemoreception is not completely understood, there is a consensus that stimuli produce membrane depolarization of glomus cells, leading to increases in [Ca^2+^]_i_ and, consequently, transmitter release (see López-López et al., 2001; Weir et al., 2005). Altogether, aforementioned evidence indicates that chemoreceptor stimuli (low PO_2_ or pH, high PCO_2_ among others) concomitantly depolarize and uncouple glomus cells, stimulating the transmitter secretion and, consecutively, increase CN chemosensory discharge. Since Cx channels are modulated by Ca^2+^ and membrane potential, it is necessary to study how these variables modify the function of GJC and/or hemichannels in glomus cells, and how those possible modifications are relevant for the chemosensory process.

Carotid bodies are involved in the response to acute and chronic hypoxia. Several studies reported that CB responses to physiological and pharmacological stimuli are enhanced after acclimation to chronic hypoxia (Rey et al., 2004; He et al., 2005, 2006). Shortly after Cx43 was described in the CB (Abudara et al., 1999, 2000), the upregulation of this protein by chronic hypoxia was reported (Chen et al., 2002). Interestingly, the augmented response may be related to the increase of transmitter release by the glomus cell (Jackson and Nurse, 1997; Eyzaguirre and Abudara, 1999), which, in turn, can be associated with a decrease in glomus cells coupling (Jiang and Eyzaguirre, 2006). All the evidence presented above are based on the chemical synapsis between glomus cells and CN endings. The identity of the synaptic transmitter was investigated (see Zapata, 1997a) and solved by Nurse’s group using an *in vitro* preparation of co-cultured glomus cell clusters and petrosal neurons. With a cocktail of suramin and hexamethonium, they blocked the hypoxic chemotransmission from glomus cells to neurons (Zhang et al., 2000). We tested the combined cholinergic-purinergic block *in situ* and and *in vitro*, but it did not prevent the hypoxia-induced increases in chemosensory discharge in the CN (Reyes et al., 2007a, b). The fact that chemoreception transmission can be blocked *in vitro*, but not *in situ*, revealed some caveats related to the cell coupling. To our knowledge, there are no results showing the effect of GJC and/or hemichannels blockers in the CB chemosensing process. The lack of GJC blockers in those experiments is relevant because Eyzaguirre’s group described dye and electrical coupling between glomus cells and CN endings (Eyzaguirre et al., 2003; Jiang and Eyzaguirre, 2006). Altogether, the evidence suggests that chemotransmission from glomus cells to CN endings may as well include electrical synapses.

It is noteworthy that coupling between glomus cells and CN endings is more complicated than the glomic coupling. First, coupling between glomus cells and CN endings presents a clear rectification. Thus, current from glomus cell spreads to nerve ending as easily as to other glomus cell, but current from nerve ending spreads poorly to glomus cell, thereby the coupling is mostly unidirectional. Also, current transmission is capacitive at the beginning and the end of the stimulus with little or no resistive component during stimulation. Additionally, CN endings are also coupled, and this specific electrical communication is capacitive and bidirectional (Jiang and Eyzaguirre, 2006). Recently, Cx36 was described in the CB, but it is unknown the cell type in which it is expressed (Frinchi et al., 2013). Considering that Cx36 has been described mainly in neuronal cells in the CNS (for review see Condorelli et al., 2000), it may be also present at the nerve endings. Thus, if neurons express mostly Cx36 and glomus cells express mainly Cx43, bidirectional communication between glomus cells and between CN endings, can be explained by the formation of homomeric homotypic GJCs. Furthermore, the formation of homomeric heterotypic GJCs between glomus cells and CN endings may explain the unidirectionality of that coupling (Jiang and Eyzaguirre, 2006). Indeed, the fact that two elements are enough to explain this phenomenon, does not exclude the putative participation of several other Cxs, currently not yet described in the CB system.

After chronic hypoxia, ventilatory or chemosensory discharge responses to different stimuli are augmented. This phenomenon may involve an enhanced release of transmitters by glomus cells, but it may also be clarified by the consideration of electrical synapses and the interaction between cells in the CB. During hypoxia, the glomic coupling is reduced. Conversely, the coupling between glomus cells and CN endings is enhanced, as well as the coupling between CN endings. This boosted coupling may allow the transmission of electrical changes from the glomus cells membrane to CN endings. Also, the enhanced coupling between nerve endings may assure the generation or multiply the action potentials in the CN, depending whether the coupled endings originate from the same neuron or from two independent neurons.

Channels formed by Cxs transmit information in another way. Recent studies show that some Cx hemichannels are permeable to Ca^2+^ (Schalper et al., 2010; Fiori et al., 2012), and it is known that chemosensory stimuli rise glomus cells [Ca^2+^]_i_ (Buckler and Vaughan-Jones, 1994a,b; Abudara et al., 2001; Jiang and Eyzaguirre, 2004; Xu et al., 2006; Lowe et al., 2013). Stimulated glomus cell may excite the secretion of a non-stimulated coupled glomus cell as a calcium wave-related second messenger, like AMPc (provided the wave occurs before the aforementioned uncoupling). Also, the same stimulated glomus cell may induce membrane depolarization of the CN endings via Ca^+2^ currents through Cx. Therefore, it could be interesting to test if Cx hemichannels are responsible—at least in part—of this phenomenon.

Bearing in mind that purinegic synapses have been considered as important components of the chemotransmission (Acker and Starlinger, 1984; Alcayaga et al., 2000; Zhang et al., 2000; Xu et al., 2003; Conde and Monteiro, 2004; Reyes et al., 2007a, b; Brown et al., 2011; Lowe et al., 2013; Piskuric and Nurse, 2013), it appears to be relevant that ATP can be released through Cx hemichannels (Kang et al., 2008). In this scenario, a stimulated glomus cell may excite neighboring cells (despite they were coupled or not with the original glomus cell) releasing ATP, which may acutely stimulate the target cell—either glomic, sustentacular or neuron—or have a chronic effect (Lin et al., 2008). However, this hypothesis has yet to be tested.

Finally, Panx-1 has been recently studied in the CB system where Type II cells were found to express this protein (Zhang et al., 2012). Type II cells are in close contact with glomus cells (McDonald and Mitchell, 1975) and some evidence suggests they may be connected via GJCs (Kondo, 2002). Also, Type II cells express metabotropic purinergic receptors, and may differentiate into glomus cells, under the adequate conditions (Pardal et al., 2007). Nonetheless, it is unclear if Type II cells do participate on the chemoreception/transmission processes. Recently, Nurse’s group reconstructed *in vitro* a tripartite CB system, using petrosal neurons, glomic cells and sustentacular—Type II—cells. In this preparation, ATP released by Type II cells, via Panx-1, stimulates neurons (Zhang et al., 2012). The three-cell model suggests that ATP released by glomus cells in response to excitatory stimulation, activates inotropic receptors at CN terminals and metabotropic receptors of Type II cells. Consequently, the [Ca^+2^]_i_ of Type II cells rises, opening Panx-1 hemichannels, which—in turn—release more ATP to the intercellular medium, thus amplifying the signal.

The functional evidence of Cxs and Panxs at the CB and their plausible participation in chemosensory transmission appears to be consistent, but more investigation on this subject is required. Additionally, it remains to be determined the presence of other types of Cx, as well as its specific location. In vitro preparations, Cxs blockers and Cxs knock-out models may clarify the physiological relevance of the intercellular coupling in the acute chemosensory process and in chronic hypoxia (sustained or intermittent).

## Cx- and Panx channels in central chemoreception

In CNS changes of CO_2_/pH are sensed by central chemoreceptors. Its location has been studied using different approaches. In mammals,* in vivo* and *in vitro* findings showed chemosensitive areas diffusely located in the brainstem, including: nucleus of solitary tract (NTS), retrotrapezoide nucleus (RTN), parafacial respiratory group (pFRG), locus coreuleos (LC), raphé nuclei and ventrolateral medulla (VLM; Elam et al., 1981; Loeschcke, 1982; Coates et al., 1993; Wang et al., 1998, 2001; Richerson et al., 2001; Messier et al., 2002; Nattie and Li, 2002a, b; Guyenet, 2008; Li and Nattie, 2008; Gargaglioni et al., 2010; Hodges and Richerson, 2010; Putnam, 2010; Ray et al., 2011; Corcoran et al., 2013; Guyenet et al., 2013). Moreover, areas related to respiratory rhythm generation—as Pre Bötzinger nucleus—also showed chemosensitivity upon exposure to CO_2_ (Solomon et al., 2000; Solomon, 2003a). Most of these studies were performed using specific blockers for predominant synapses in each preparation; glutamatergic and GABAergic blockers; or synaptic blockade medium (high Mg^2+^-low Ca^2+^). However, none of those experiments included GJC blockers, so it is possible that chemosensory nuclei could be less responsive.

**Figure 3:**
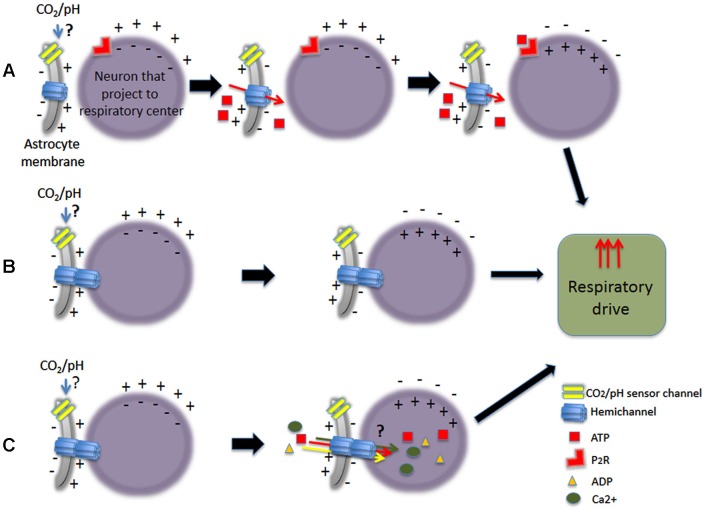
**Schematic representation of some of the different pathways proposed upon CO_2_-dependent stimulation of astrocytes increasing respiratory drive**. Astrocytes could stimulate neurons that project to respiratory center, being either chemosensory neurons or not. **(A)** Chemical stimulation by ATP release. **(B)** Stimulation by electrical coupling. **(C)** Stimulation of neurons by electrical coupling with passage of small molecules.

As it is well known, hemichannels participate in diverse functions of CNS (reviewed by Menichella et al., 2003; Kielian, 2008; Kleopa et al., 2010; Abrams and Scherer, 2012; Mika and Prochnow, 2012; Belousov and Fontes, 2013). Since Cxs proteins are expressed in several CNS regions involved in central chemoreception, it is possible that hemichannels may play a role in pH/CO_2_ sensing. They could increase the neuronal response in chemosensory nuclei and/or directly sense the hypercapnic stimulus. For many years, pH/CO_2_ central chemosensing has been described as a property restricted to neurons, discarding that astrocytes could sense pH/CO_2_. Now, if hemichannels are involved in direct chemosensing, it would implied that astrocytes could also be considered as chemoreceptors (Figure 3).

At the moment, more than 15 Cxs isoforms are described in the rodent brain (Dermietzel and Spray, 1993; Condorelli et al., 1998), but the first study showing electrotonic coupling between neurons of mammalian brainstem predates all descriptive ones (Llinás et al., 1974). Later, different researchers showed that Cx26, Cx30, Cx32, Cx36 and Cx46 are predominant in the brain with different cellular distribution. While Cx32 and Cx36 are principally expressed in neurons, Cx30 and Cx43 are mainly expressed in astrocytes, and both cell types share Cx26 (Dermietzel et al., 1989; Yamamoto et al., 1990; Nagy et al., 1997, 1999, 2001; Condorelli et al., 1998; Rash et al., 2001).

Cx26, Cx32 and Cx36 are expressed in rat putative chemosensory nuclei, such as RTN, raphe, LC and Pre Bötzinger (Alvarez-Maubecin et al., 2000; Solomon et al., 2001; Solomon, 2003b). Furthermore, mRNA of Cx36 and Cx43 have been identified in the ventral respiratory group and in XIIn, respectively (Parenti et al., 2000). GJCs were reported in the dorsal aspect of the medulla oblongata showing electric and anatomical coupling in dorsal nucleus of the vagus (DMV) and NTS, during and after at least one exposure to hypercapnic acidosis (Dean et al., 1997, 2002; Huang et al., 1997).

On the other hand, the molecular mechanism of central chemoreception has not been well established yet. Until now, there are several pH-sensitive K^+^ channels considered as candidates (Wu et al., 2004; Yuill et al., 2004; Zhang et al., 2006; Lazarenko et al., 2010; Wenker et al., 2010; Huckstepp and Dale, 2011; Hawryluk et al., 2012; Wang et al., 2013b). Also, ATP appears to be involved. It is well known that in peripheral sensory neurons both, ATP receptors—ionotropic P_2_X or metabotropic P_2_Y—excite afferent fibers. Therefore, ATP contributes significantly to the CB chemotransmission, being released by chemoreceptor cells and thus activating sinus nerve endings (Alcayaga et al., 2000; Rong et al., 2003 and reviewed by Spyer et al., 2004).

Bearing in mind the contribution of ATP in peripheral chemotransmission, its participation has been studied in the central chemoreception. Inspiratory and pre-inspiratory neurons of VLM only expresses the P_2_X_2_ receptor subunit and its activity was increased by ATP and blocked by suramin (Gourine et al., 2003). In order to accurately measure real time changes of ATP concentration, Gourine group developed a microelectrode biosensor detecting an almost immediate release of ATP upon CO_2_ stimulation in rats. Using horizontal slices of medulla oblongata, they detected a marked release of ATP from the most ventral slice (mainly from RTN), upon CO_2_-induced acidification of the incubation media. Moreover, blocking ATP receptors at these sites diminishes the chemosensory control of breathing. During hypercapnia, the increase in ATP release occurred 19.5 ± 4.8 ms before the induction of breathing. Based on above evidence, they hypothesized that ATP-mediated afferent transduction may also occur in the central chemoreception (Spyer et al., 2004; Gourine et al., 2005), as is described in peripheral chemoreception (Prasad et al., 2001; Zapata, 2007; Piskuric and Nurse, 2013).

In adult rats, recordings of respiratory activity of phrenic nerve showed that bilateral injections—at RTN level—of a P2 receptor blocker decreased by 30% the ventilatory responses to CO_2_. Conversely, the inhibition of P_2_Y_1_ receptor—at the same level—had no effect on CO_2_ responsiveness neither *in vitro* nor *in situ* (Wenker et al., 2012). Taking together, these results indicate that modulation of P_2_X_2_ receptor function (e.g., during hypercapnia) may contribute to changes in the activity of the VLM respiratory and chemosensory neurons that express those receptors. Interestingly, P_2_X_2_ and P_2_X_3_ receptor subunits knock-out mice have normal ventilatory response to hypercapnia (Rong et al., 2003 and reviewed by Erlichman et al., 2010).

The CO_2_-dependent ATP release persisted in the absence of extracellular Ca^2+^, i.e., it did not occurred via neuronal exocytosis. This release—presumably from astrocytes in ventral surface of rat brainstems—depends on hemichannels formed by Cx26. Additionally, three different methods showed that HeLa cells expressing Cx26 release ATP in response to CO_2_ (whole cell patch-clamp, CO_2_-dependent dye uptake and patch clamp “*inside-out* and *outside-out*”). In HeLa cells model, changing PCO_2_ from 35–70 mmHg evokes outward currents, increases the current noise, and also causes rapid and large increases of the conductance. The gating of Cx26 hemichannel increased and decreased in response to increases and decreases of PCO_2_, respectively. Interestingly, only Cx30 and Cx32 (classified as β Cxs), exhibited sensitivity to changes in PCO_2_ (Huckstepp et al., 2010a, b). This evidence indicates that astrocytes (additionally to neurons) could be considered as chemoreceptors in the CNS, and it also suggests that Cxs are sensors for the extracellular CO_2_/pH (reviewed by Funk, 2010). Recently, evidence demonstrate CO_2_ binding to Cx26, and that this interaction was probably via carbamylation of K125 motif. The authors hypothesized that CO_2_ would form a carbamate bridge between the K125 of one subunit and the R104 of the adjacent subunit, therefore opening the Cx26 hemichannel (Meigh et al., 2013).

An alternative hypothesis proposed that astrocytes would be pH-sensitive. This notion derived from *in vitro* studies of RTN, a specific area within VLM. The sensitivity expressed as pH-sensitive currents involved either, Kir4.1-Kir5.1 channels and/or sodium/bicarbonate cotransporter (Wenker et al., 2010). Moreover, removal of pia matter irreversibly eliminates CO_2_-evoked ATP release, indicating the importance of structural integrity of the marginal glial layer of the ventral medullary surface. Based on these observations, the marginal glial layer appears to be the likely source of ATP release in response to CO_2_/pH (Spyer et al., 2004; Erlichman et al., 2010).

Many putative chemosensory nuclei in the medulla oblongata are ATP-sensitive areas, including RTN, raphé nuclei and LC. As mentioned previously, just a few of them have been studied more thoroughly pointing out the possible involvement of ATP and astrocytes in central chemoreception.

At the LC, the participation of ATP in the central chemosensory mechanism is supported by ATP-induced neuronal depolarization. This depolarization was reduced by 30 mM suramin and abolished by 100 mM suramin. In addition, suramin potentiated the excitatory AMPA effect, but did not alter the inhibitory effect of noradrenaline (Nieber et al., 1997). It remains to be elucidated where ATP is released from, astrocytes, neurons or both. It is unclear if ATP is released as the sole transmitter from purinergic neurons projecting to LC. Also, it is uncertain if ATP is released as co-transmitter with noradrenaline from recurrent axon collaterals—or dendrites—of LC neurons themselves. Finally, the LC responded to CO_2_ with synchronic activity maintained in spite of synaptic blockade (Andrzejewski et al., 2001). This may be explained considering the expression of Cx at the LC (Solomon, 2003a) which, as previously mentioned, may be involved in the chemosensory activity.

Early studies in the ventral medulla showed that cells with electrophysiological characteristics of astrocytes depolarized during hypercapnic condition (Fukuda et al., 1978) Many years after, Gourine group (Gourine et al., 2010; Kasymov et al., 2013) demonstrated that astrocytes from VLM responded to physiological acidity with important increases in intracellular Ca^2+^ and release of ATP. Also, they mimic Ca^2+^ responses evoked by pH, using optogenetic stimulation of astrocytes expressing channelrhodopsin-2. Thus, activating chemoreceptor neurons via ATP-dependent mechanism and triggering robust respiratory response *in vivo*, demonstrated a potential role of brain glial cells in central chemoreception. Cx expressed in astrocytes were related to Ca^2+^ waves, which have been involved in intercellular transmission of information (reviewed by Scemes and Giaume, 2006). Recently, the direct demonstration of Ca^2+^ flux through purified Cx26 hemichannels reconstituted in liposomes, suggested that Ca^2+^ fluxes through hemichannels can be a pathway for Ca^2+^ influx into cells in physiological and pathological conditions (Fiori et al., 2012). Hence, astrocytes could stimulate adjacent neurons by releasing ATP through hemichannels and also by Ca^2+^ waves through GJCs.

In summary, the evidence revisited here indicates that astrocytes may have a preponderant participation in central chemoreception. They respond to CO_2_/pH increasing their intracellular Ca^2+^ levels and releasing ATP by mechanisms still unknown that may include Cxs (at least Cx26). Released ATP would excite ATP-sensitive neurons that directly innervate the respiratory controller. Most of other chemosensory areas are ATP-sensitive and express Cxs (potentially forming functional hemichannels). Therefore, ATP and Cxs could be part of a common mechanism in chemosensory nuclei. Considering the evidence, these mechanisms may occur at RTN, but further studies are required to demonstrate the participation of Cxs in other chemosensitive areas.

Finally, despite the knowledge that Panx are expressed in the brain (Panchin et al., 2000; Bruzzone et al., 2003; Baranova et al., 2004), their functional expression in central chemosensory areas has not been studied so far. Panx could be participating in chemosensory processes in a similar way than Cxs do.

Reviewing the abovementioned studies, there is evidence to enlighten the central chemoreception. However, several questions arise about the cellular identity of the chemoreceptor and the signaling pathways involved.

Firstly, is there an overestimation in the number and/or types of chemosensory cells? Considering the absence of GJCs blockers in chemosensory recordings, if a non-chemosensitive cell is coupled to a chemosensitive cell, the first one will also present chemosensory responses to CO_2_. This may lead to an overestimation of the chemosensitive cells population. Also, the overestimation may be due to the effect of ATP released from a chemosensitive cell (neuron or astrocyte) exciting neighboring non-chemosensitive cells (neurons and/or astrocytes), which in turn will be considered as chemosensitive cells. As it now appears, the release of ATP in response to CO_2_ may involve Cx or Panx hemichannels. Secondly, if astrocytes are also chemoreceptors, do they have different sensitivity to CO_2_/pH than that of neurons? If neurons are more sensitive, they will respond to lower changes in CO_2_/pH, and then the astrocyte response may increase/potentiate/synchronize the nucleus response. If astrocytes are more sensitive than neurons, they may prime chemosensitive neurons, which directly innervate the respiratory controller. Thirdly, do neurons and astrocytes share a common mechanism of CO_2_/pH sensing? Are there multiple mechanisms involved? It seems like Cxs could be sensing CO_2_ or pH—as many pH—sensitive K^+^ channels, but neurons express both. Therefore, there are many facts still pending to be clarify. Fourthly, are the ATP-sensitive chemosensory areas also sensitive to other transmitters released by astrocytes? It is known that astrocytes release ATP, but they also release adenosine that may as well be involved in the excitation of neighboring cells. Finally, the presence of Cxs and Panx in the chemosensory system may represent an alternate—independent—via to increase the response to hypercapnia.

## Conflict of interest statement

The authors declare that the research was conducted in the absence of any commercial or financial relationships that could be construed as a potential conflict of interest.
